# Assessing reward preference using operant behavior in male and female mice

**DOI:** 10.1371/journal.pone.0291419

**Published:** 2023-09-12

**Authors:** Rose-Marie Karlsson, Heather A. Cameron

**Affiliations:** Section on Neuroplasticity, National Institute of Mental Health, National Institutes of Health, Bethesda, Maryland, United States of America; Kyoto University Graduate School of Informatics: Kyoto Daigaku Daigakuin Johogaku Kenkyuka, JAPAN

## Abstract

Many different solid food pellets are available as reinforcers for rodents in operant behavior tests. Different reward formulations have not been compared, so it is unclear whether mice show strong preferences for different rewards and whether such preferences are consistent within or across sex and background strain. Here we show that mice have strong preferences for two balanced diet food rewards over sucrose pellets, and preference for one balanced diet pellet formulation over another, in a simultaneous choice test using a low effort fixed ratio operant test. All mice, of both sexes and both CD1 and C57 background strains, showed the same strong preferences among these three types of reinforcers. In contrast, flavorings added to the reward pellets had relatively small and more variable effects on preference. The preference for balanced diet pellets over sucrose pellets was seen also in the total numbers of rewards consumed in low effort tests with food pellets or only sucrose pellets available. However, progressive ratio testing showed that mice worked harder for sucrose pellets than for the preferred balanced diet pellets. These findings indicate that reinforcers with similar and very different preference profiles are readily available and that testing with different rewards can produce different, and sometimes unexpected, results.

## Introduction

Operant conditioning, which rewards or punishes particular behaviors, is an important and frequently used behavioral paradigm in neuroscience research. The flexibility of operant testing provides opportunities for a wide variety of conditions, allowing many different behaviors to be tested and providing multivariate data from large numbers of trials in a fully automated fashion. Reinforcers are necessary to motivate animals to work in an operant setting. Aversive stimuli can be used as negative reinforcement, but operant conditioning often uses reward-based motivation, generally water or a caloric food or liquid to water- or food-restricted animals, respectively. A wide variety of caloric rewards are available, including sweetened water or milk-based liquids and many types of easily-dispensed commercially-available food pellets.

The choice of a particular reward is often somewhat arbitrary and given little thought, as long as it drives strong enough motivation for animals to perform the required task. However, a recent operant conditioning study from our laboratory found that mice and rats lacking adult hippocampal neurogenesis showed decreased motivation in two tasks when working for chocolate flavored sucrose rewards but normal behavior when working for balanced diet rewards [1]. Further testing in a simple operant free choice test showed a strong, somewhat unexpected, preference for the balanced diet rewards relative to the sugar rewards, suggesting that the motivation effect of the new neurons occurred only with low value rewards. Our results regarding relative reward preference and the importance of reward strength for understanding behavior in this paradigm led us to seek additional information regarding preferences and effort expended for different commonly available palatable food rewards.

Information on relative reward strength of various pellets should be useful for planning and interpretation of any operant behavior study and may be particularly valuable for investigating motivation and feeding behaviors. Recent studies have compared the strength of various liquid reinforcers in operant paradigms [2–4], or compared liquid to pellet rewards [5]. However, the reinforcing properties of solid food rewards has been systematically compared in pigeons but not rodents [6]. Here we use predictable, low effort (FR1) simultaneous two-lever choice preference tests as well as sequential single lever progressive ratio (PR) tests in mice to compare commercially-available sucrose reward pellets and two different formulations of balanced diet pellets, each in their plain form or with added flavorings. Because previous studies have found strain differences in performance across different rewards [5], and no studies have examined reward preference in female mice, we tested males and females from two different mouse strains. We find strong and consistent preferences in preference tests, which varied little across mouse sex and background strain, with relatively small but counterintuitive effects in PR tests.

## Materials and methods

### Animals and general procedures

All procedures were approved by the Animal Care and Use Committee of the National Institute of Mental Health. Male and female CD1 and C57BL/6J (C57) mice were obtained from Charles River (Raleigh, NC) and The Jackson Laboratory (Bar Harbor, ME, USA), respectively, at seven weeks old. Mice were group housed (4/cage), separated by strain and sex, under a reversed light cycle (lights off at 8:00 a.m.) with ad lib LabDiet Prolab RMH 1800B diet. After 5 days of habituation, mice were food restricted to reach 85–90% of their free feeding weight. After 6–7 days of food restriction, operant training began at 10:00 am each day. Each mouse was tested in only one comparison or preference testing experiment.

### Apparatus

Testing was done in operant chambers (Med Associates, St. Albans, VT, USA) enclosed in sound attenuating enclosures. Each chamber was equipped with two retractable levers, each of which was connected to a separate pellet dispenser. A single reward magazine was located between the levers. The fan was turned on to signal the start of a training session during which lever responses activated the associated pellet dispenser. Med-PC V (Med Associates, St. Albans, VT, USA) controlled and recorded lever presses and reward deliveries.

### Reward pellets

All tests used 14 mg reward pellets. We used three different basic reward pellet formulations: 1) Sucrose rewards (“sucrose”, item# 5TUT, TestDiet, St Louis, MO) contained sucrose, dextrose, magnesium stearate, and inert binding and were either plain or had banana, chocolate, or peanut butter flavoring added. Nutritionally, they contained 85.1% carbohydrates and 4.7% fiber and had a calorie content of 3.40kcal/g. 2) Purified Rodent Tablets (“PRT”, item# 5TUL, TestDiet, St Louis, MO) are a balanced diet reward containing sucrose, casein, maltodextrin, corn starch, corn oil, cellulose, minerals, silicon dioxide, vitamins, magnesium stearate, DL-methionine, either plain or with banana or chocolate flavoring added. Nutritionally, they contained 57.5% carbohydrates, 17.7% protein, 4.9% fat and 4.8% fiber with a calorie content of 3.44kcal/g. 3) Dustless Precision Pellets (“DPP”, item# F05684, Bio-Serv, Flemington, NJ) are a balanced diet pellet containing sucrose, dextrose, casein, corn oil, mineral mix, cellulose, corn syrup, calcium silicate, vitamin mix, magnesium stearate, choline bitartrate, DL-methiononem L-cyctein, ascorbic acid, vitamin E acetate and were all plain, with no added flavoring. Nutritionally, they contain 59.1% carbohydrate, 18.7% protein, 5.6% fat, 4.7% fiber and have a calorie content of 3.60kcal/g.

### Reward preference testing

This experiment was conducted to determine whether mice had different intrinsic preferences for particular reward pellets and whether these preferences were consistent across sex and strain. Each of the two pellet dispensers contained a different type of 14 mg reward pellet. Training began with a 20 min session during which 20 pellets (10 rewards of each flavor) were made available in the magazine. After the first session, mice were trained to associate a single lever (left or right) with a particular reward on a Fixed Ratio 1 (FR1) schedule, with a reward given for every 1 lever press, and an endpoint of 30 rewards or 30 min. FR1 training continued for 6 daily sessions, 3 alternating sessions for the left and 3 for the right lever. After these acquisition sessions, the animals received 6 daily 30-min sessions in which they had access to both levers and were able to press to deliver both rewards on an FR1 schedule as before. The first 4 sessions were treated as habituation/stabilization sessions, and the last 2 sessions were analyzed and averaged. Three different experiments were run with naïve mice in each experiment. Experiment 1 compared the two different balanced diet pellets to each other and to sucrose pellets. Experiment 2 compared plain 5TUL pellets to PRT pellets with added flavorings. Experiment 3 compared plain sucrose pellets to sucrose pellets with added flavorings. Analyses in these direct preference experiments were unpaired t tests comparing consumption for directly tested pairs of rewards. Effects of sex and strain on total pellet consumption were compared using two-way ANOVA with sex and strain as factors and two-way ANOVA with strain and reward type as factors (in males only).

### Progressive ratio testing

Training began with a 20 min session with 20 pellets made available in the magazine. The animals then underwent training on fixed ratios (FR) before advancing to progressive ratio (PR) testing to assess motivation. Mice were first trained to lever press for rewards on an FR1 schedule, with one active and one inactive lever available and an endpoint of 30 rewards or 30 min. After the FR1 training, mice then moved on to 3 sessions each of FR3 and FR5, with mice receiving rewards for every 3 or 5 lever presses, respectively. Following this training, mice responded under a PR schedule where the reinforcement was contingent on the following ratio progression: 1, 2, 4, 6, 9, 12, 15, 20, 25, 32, 40, 50, 62, 77, 95, 118, 145, 178, 219, 268, 328, 402, 492, 603, etc. [7, 8], which reset each session. Breakpoints were defined as the number of ratios completed (i.e., number of rewards delivered), allowing up to 20 min to earn each reinforcement. PR was tested for 5 consecutive days; the first 3 were considered as habituation sessions, and the last 2 sessions were analyzed. Separate cohorts of male mice were used to test motivation for sucrose or PRT balanced diet rewards, both plain/unflavored. Break points, overall session time, lever pressing rate, and head entry rate were analyzed using two-way ANOVA with reward type and strain as factors.

## Results

### Balanced diet versus sucrose preference

**Fig 1:**
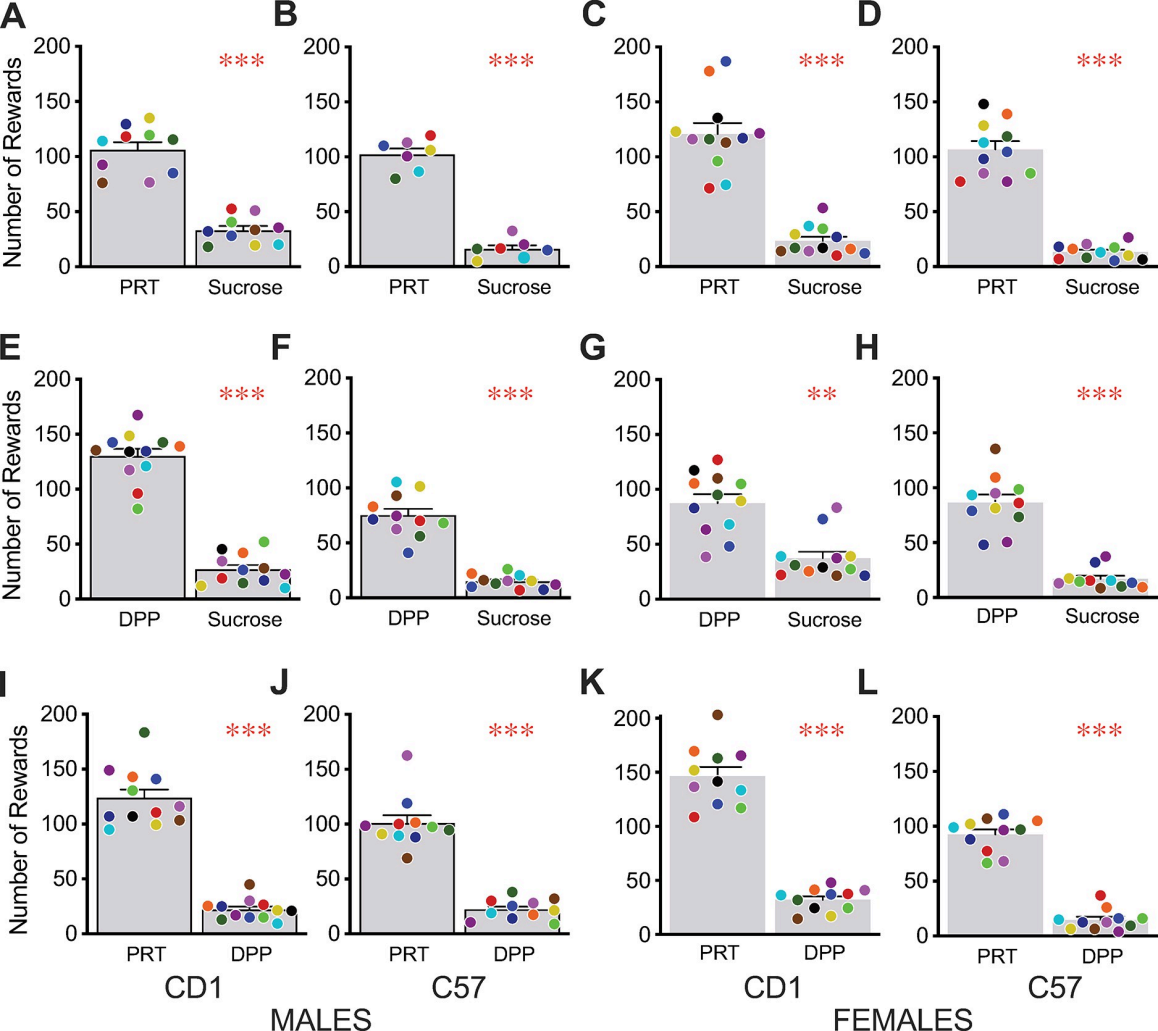
Direct preference testing of sucrose and balanced diet pellets. CD1 male (A,E,I), C57 male (B,F,J), CD1 female (C,G,K), and C57 female (D,H,L) mice were given access to different pairs of reward pellets on an FR1 schedule. All mice, regardless of sex or strain, consumed more Purified Rodent Tablets balanced diet rewards (PRT, TestDiet) than Dustless Precision Pellets balanced diet rewards (DPP, Bio-Serv) or sucrose pellets and more DPP pellets than sucrose. Bars reflect mean ± SEM, dots represent individual mice with the same color signifying the same mouse for both choices, ** p < 0.01, *** p < 0.001.

We observed a significant preference for the TestDiet balanced diet rewards (PRT) over sucrose pellets in CD1 male mice, as previously reported [1]. This strong preference was also found in male C57 mice as well as females of both strains (Fig 1A–1D). The other balanced diet food reward, Dustless Precision Pellets Bio-Serv (DPP), was also strongly preferred over sucrose rewards across both strains and sexes (Fig 1E–1H). Lastly, we assessed the preference for the two different balanced diet rewards and found that both strains and sexes strongly and significantly preferred the PRT pellets to the DPP pellets (Fig 1I–1L). Taken together, these tests showed three distinct levels of preference, with PRT > DPP > sucrose pellets. These preferences were strong and shared by every mouse tested without exception.

### Preference testing for different food flavors

**Fig 2:**
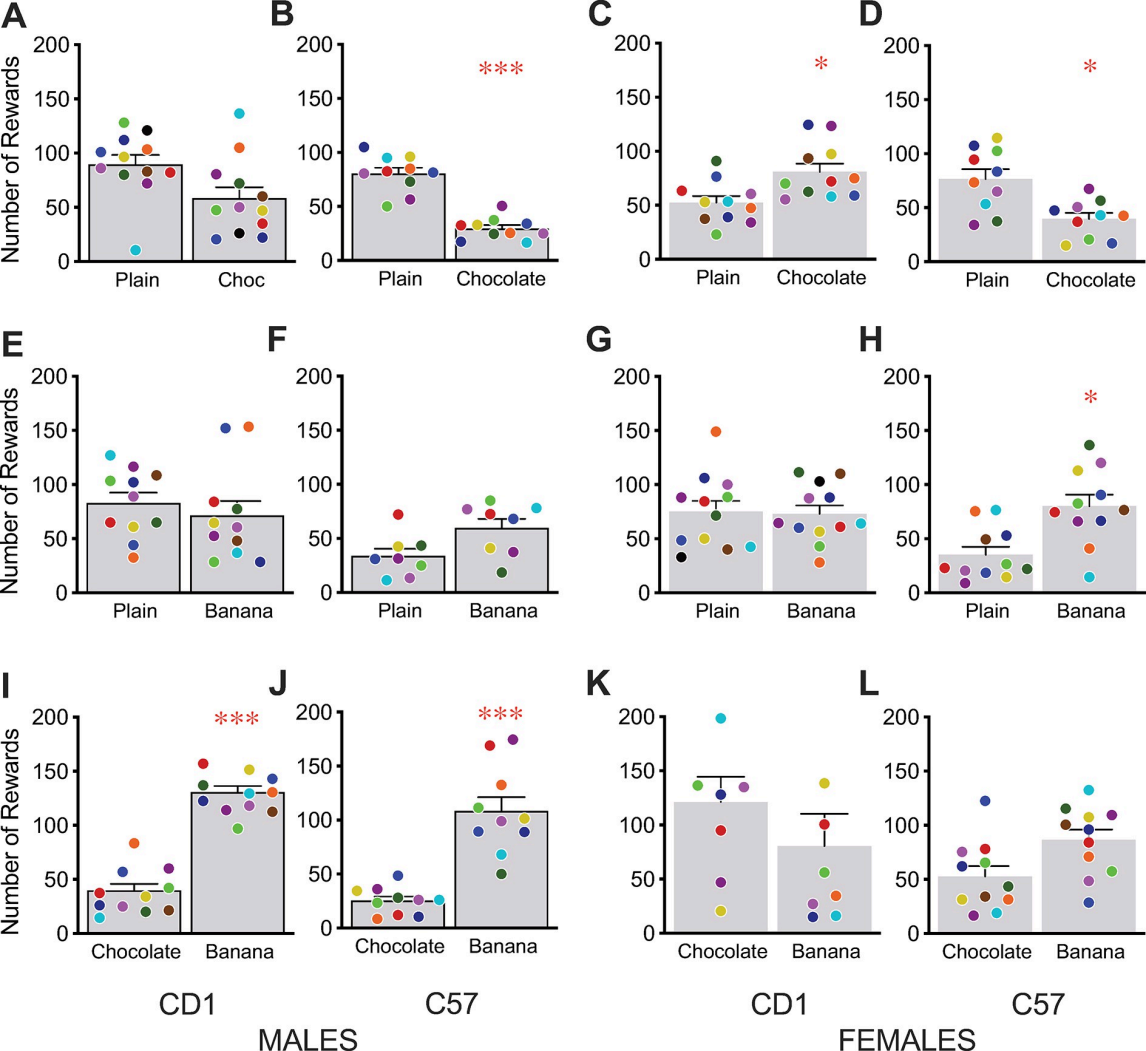
Direct preference testing of different flavors of balanced diet pellets. CD1 male (A,E,I), C57 male (B,F,J), CD1 female (C,G,K), and C57 female (D,H,L) mice were given access to pairs of PRT reward pellets with different added flavorings on an FR1 schedule. Flavor preferences were variable across, as well as within, sex and strain. Bars reflect mean ± SEM, dots represent individual mice with the same color signifying the same mouse for both choices, * p < 0.05, *** p < 0.001.

Balanced diet reward pellets come in flavored versions as well as the plain/unflavored versions tested above. We therefore compared 3 different PRT flavors: plain, chocolate, and banana flavor. C57 mice, both males and females, showed a significant preference for the plain food rewards compared to the chocolate-flavored food rewards, whilst the CD1 mice showed no preference in this pairing (Fig 2A–2D). Banana-flavored rewards were significantly preferred over plain rewards by C57 female mice (Fig 2H), but neither the C57 males nor CD1 mice showed any difference in preference (Fig 2E–2G). When comparing chocolate- and banana-flavored food rewards, males of both strains showed a significant preference for the banana-flavored rewards (Fig 2I and 2J), while the females showed no preference for either reward (Fig 2K and 2L). Putting together these pairwise preferences suggests that for C57 males, banana = plain > chocolate; for C57 females, banana > plain > chocolate (but banana = chocolate, which is seemingly inconsistent); for CD1 males, banana = plain = chocolate (but banana > chocolate); and for CD1 females, banana = plain = chocolate. Overall, the preference for particular flavors were less strong and more variable than the preferences between the different types of plain/unflavored reward pellets above.

### Preference testing for different sucrose flavors

**Fig 3:**
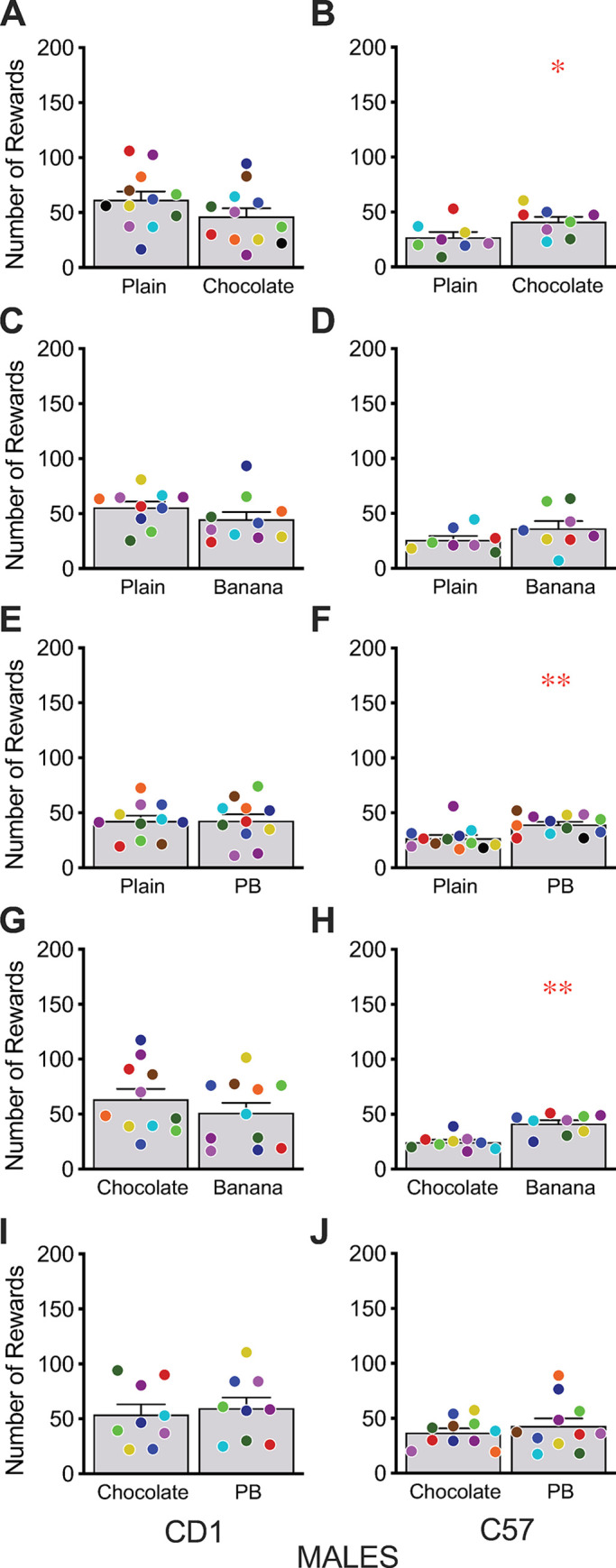
Direct preference testing of different flavors of sucrose pellets. CD1 (A,C,E,G,I) and C57 (B,D,F,H,J) male mice were given access to pairs of sucrose reward pellets with different added flavorings on an FR1 schedule. C57 mice showed weak preferences for some flavors over plain sucrose, while CD1 mice showed no significant preferences. Bars reflect mean ± SEM, dots represent individual mice with the same color signifying the same mouse for both choices, * p < 0.05, ** p < 0.01.

Testing of different sucrose flavors was conducted with five different flavorings but only in male mice. CD1 males did not show any preference for any of the sucrose flavors (Fig 3A, 3C, 3E, 3G and 3I) whilst C57 mice showed small but statistically significant preferences for chocolate- (Fig 3B) and peanut butter- (Fig 3F) flavored sucrose rewards over the plain sucrose and for banana-flavored sucrose over chocolate-flavored sucrose rewards (Fig 3H). As with balanced diet PRT pellets, flavorings in sucrose had relatively small and variable effects on preference in direct comparison testing.

### Effects of strain and sex on total pellet consumption in preference tests

**Fig 4:**
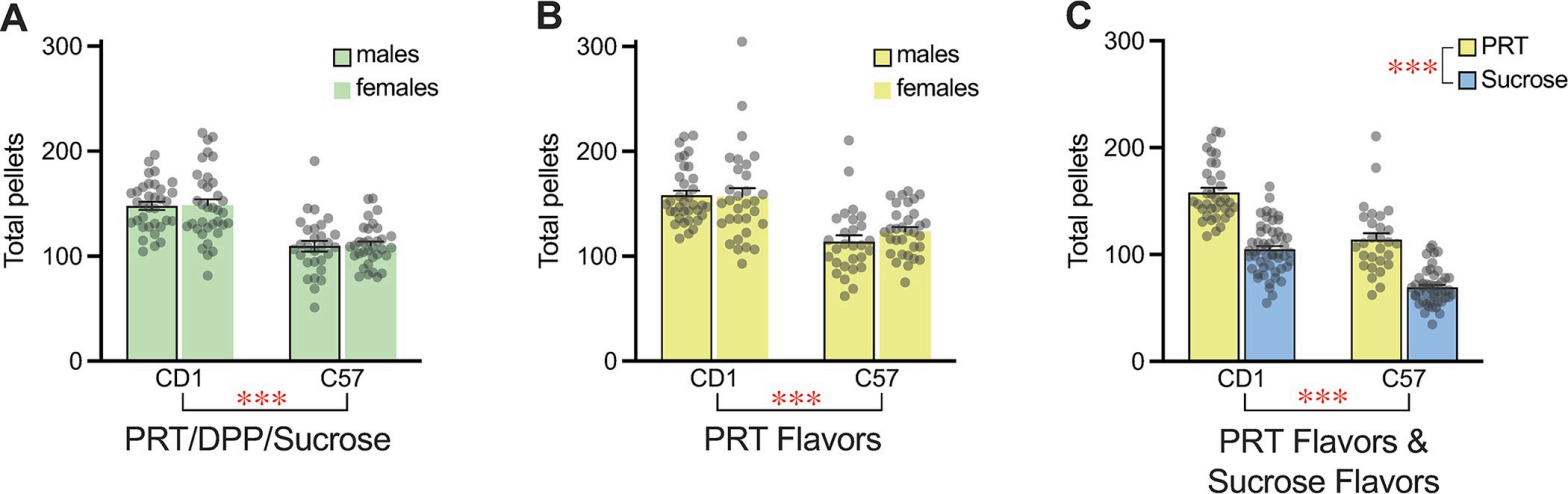
Overall pellet consumption in direct preference tests. (A) Total pellet consumption in tests with all pellet pairs from the experiment comparing sucrose with balanced diet rewards shows an effect of strain but no effect of sex on pellet consumption. (B) Total pellet consumption in all tests comparing different flavors of PRT shows an effect of strain but no effect of sex on pellet consumption and similar consumption to the initial test. (C) Total pellet consumption in tests comparing all flavors of PRT with all flavors of sucrose in males shows decreased consumption in both strains in tests with sucrose pellets as the only option relative to tests with balanced diet pellets as the only option. Bars reflect mean ± SEM, dots represent individual mice, *** p < 0.001 for main effect of strain or reward type.

When the total numbers of consumed pellets were compared across sex and strain for each experiment, irrespective of the particular pair of rewards tested, CD1 mice earned and ate significantly more rewards than C57 mice in all three preference experiments as demonstrated by main effects of strain in two-way sex x strain ANOVAs (Fig 4A–4C). Post hoc testing showed significant strain differences in both males and females (Fig 4A and 4B). Somewhat surprisingly, there was no difference in pellet consumption between males and females of either strain (Fig 4A and 4B), despite the pronounced sex difference in weight between the age-matched animals.

When pellet consumption was compared across experiments, fewer pellets were consumed in the tests in which only sucrose pellets were offered compared to the experiment comparing different flavors of balanced diet pellets in both strains (Fig 4C). This difference suggests that mice are less motivated to work for sucrose pellets, or become satiated more quickly with sucrose pellets, than with balanced diet pellets.

### Progressive ratio testing

**Fig 5:**
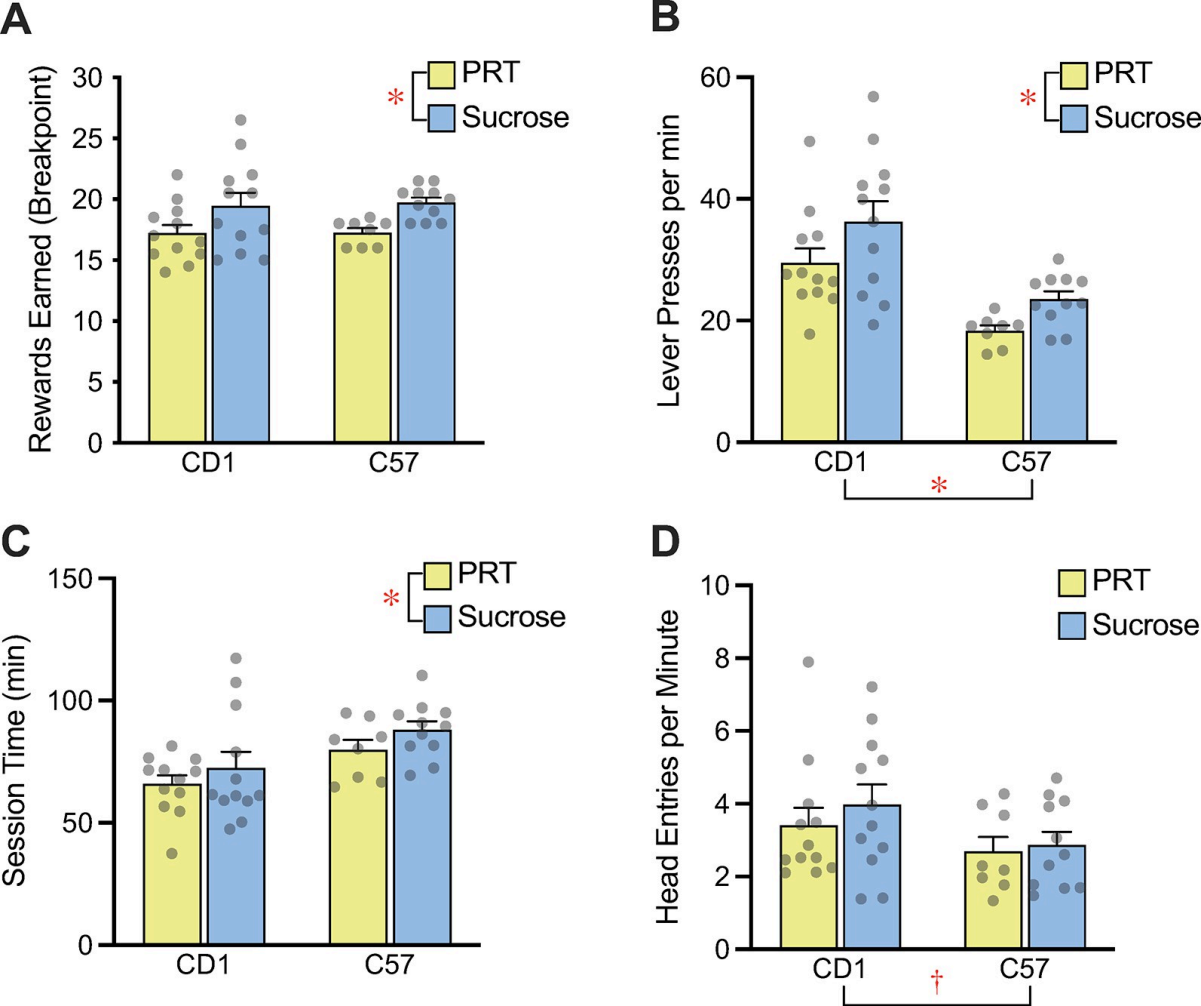
Progressive ratio testing. (A) Male CD1 and C57 mice run on a progressive ratio (PR) test for either sucrose pellets or PRT balanced diet pellets showed higher break points when working for sucrose. (B) CD1 mice show faster pressing rates than C57 mice, but both strains press faster for sucrose than PRT pellets. (C) Session length, determined by time out period of X mins, was longer when working for sucrose pellets than for PRT pellets. (D) CD1 mice had a higher head entry rate than C57 mice, but there was no effect of pellet type on this measure. Bars reflect mean ± SEM, dots represent individual mice. ** p < 0.01, *** p < 0.001, † p = 0.06 for main effect of strain or reward type.

To further investigate reward strength, or motivation to work for different rewards, male mice of the two strains were tested on a progressive ratio with different cohorts receiving either TestDiet plain balanced diet rewards or plain sucrose reward. Surprisingly, given the strong preference for, and greater consumption of, balanced diet pellets in preference testing, both strains worked harder for sucrose pellets than for food pellets, earning approximately 2 additional sucrose rewards (Fig 5A). In addition, mice of both strains pressed faster (Fig 5B) and worked longer (Fig 5C) for sucrose pellets than for balanced diet pellets. CD1 mice had significantly higher lever press rates than C57s, but there were no effects of strain on break point or session time.

## Discussion

Our simple operant conditioning task comparing multiple commercially-available solid food reinforcers showed that mice of both sexes and strains strongly preferred balanced diet rewards over sucrose rewards. Added flavorings had relatively small and more variable effects on preference. All mice also preferred one particular formulation of balanced diet rewards, TestDiet PRT pellets, over another pellet with similar nutritional value, BioServ food reward. Surprisingly, given their strong preferences for balanced diet pellets, both strains worked harder for sucrose rewards than for balanced diet pellets in progressive ratio testing.

Rodents, like humans, prefer sweet tasting foods and liquids to non-sweet versions–in contrast to cats, which lack receptors for sweet taste [9]. Several common inbred mouse strains, including BALB/cJ and DBA/2J, carry an allele of the Tas1r3 sweet receptor that makes them “sub-sensitive” to sweetness relative to C57Bl/6, CD-1 and other “sensitive” strains. Interestingly, although this sensitivity alters preference of naïve mice for low concentration sucrose solutions, it does not affect motivation to work for moderate concentrations of sucrose or for previously sampled low concentration sucrose [10, 11]. Mice from all strains will work for sucrose rewards. However, we have found, here and in a previous study [1], that balanced diet food pellets are strongly preferred to pure sucrose. This should perhaps be unsurprising, as the balanced diet pellets combine sugar and fat, a highly rewarding combination [12]. Dietary protein is likely sufficient for the food-restricted adult mice fed on a diet containing 18% protein, but it is possible that the protein in the balanced diets, and lacking from the sucrose pellets, could play a role in reward preference as well.

Calorie content is an influential determinant of reward strength [2, 6]. However, the current study found that mice showed very strong and consistent preferences among three approximately isocaloric solid rewards, with a slightly lower calorie reward (TestDiet balanced diet) being preferred over a slightly higher calorie pellet (Bioserv). This is clear evidence that factors other than caloric density, sugar, and fat content can drive preferences in mice, though the specific properties driving this preference are not known. Added flavorings had some effect on reward preference, though these flavor preferences were relatively small and highly variable.

No previous studies that we are aware of have compared food reward preferences in female mice. We found that strong preferences, such as those between the three plain (unflavored) reward types used here, were the same in males and females. The preferences for added flavors were variable across strain and sex as well as across animals within a given strain and sex. Interestingly, despite a relative large weight difference between the age-matched male and female mice, the numbers of pellets consumed in the simultaneous free choice tests were very similar.

Reward preference and relative reward strength can be measured in several different ways, and the relationship between these parameters are not always straightforward. Few studies have directly compared rewards in simultaneous choice tests. One such study [6] compared reward preferences in pigeons by presenting three different freely available rewards at a time and comparing the amounts of each that were eaten (by weight). The current study, as well as our previous one [1], used a modified version of this simultaneous choice approach, requiring mice to press one of two levers to receive a corresponding reward but on a very low-effort FR1 schedule, with both showing that mice have strong preferences for the balanced diet tablets over sucrose pellets.

Virtually all previous studies comparing consummatory rewards in mice have assessed rewards according to their ability to motivate effort in a PR task. These studies have shown that mice work harder, with increased response speed and/or break points, for larger rewards [13], high fat or high sugar milk versus low fat milk [2]; and sweetened milk relative to sweetened water [3]. Although none of these studies tested their rewards in a direct comparison, the more strongly motivating rewards in both of these studies seem likely to be preferred in simultaneous choice tasks. It is therefore somewhat paradoxical that in the current study we found that despite the strong preference for balanced diet pellets over sucrose in simultaneous choice tests, both mouse strains worked harder for sucrose in the PR task than for the strongly preferred TestDiet pellets. PR testing for the two rewards was done in separate cohorts of naïve mice, so testing order and successive contrast effects cannot explain this finding. In addition, although none of the mice used for PR testing underwent simultaneous preference testing, every mouse in that test strongly preferred the TestDiet pellets, suggesting the mice in the PR test would as well. In a previous study, we found the same preference for TestDiet pellets over sucrose pellets in a simultaneous choice test and ran PR tests with each of these rewards [1]. These published PR tests for different rewards were run at different times in different cohorts of mice, but if they are directly compared, they also suggest stronger motivation to work for sucrose pellets in control CD-1 mice.

The reasons for this disagreement between preference and motivational strength are unclear. One possibility is that differences in the speed or effects of metabolizing the two reinforcers, due to their different nutritional profiles, affect animals’ work to earn the pellets. Alternatively, there may be a cognitive explanation related to the different amounts of effort and uncertainty involved in the two tests. High effort can increase palatability of rewards and does so more for rewards that are initially less-preferred reinforcers than for those that are more palatable [14, 15]. This suggests that PR testing could potentially increase the preference for sucrose reward pellets without increasing palatability of the TestDiet pellets, though we are not aware of examples in which reward preference switched following effort expenditure. Alternatively, the apparent paradox in working harder for the less preferred reward could be an example of “irrational wanting” or difference between “liking” and “wanting” [16, 17].

Taken together, these findings demonstrate that commercial reward pellets are available in at least three distinct levels of preference that are consistent across sex and at least two different strains of mice. These preferences predict total consumption in a low effort test, but they do not predict effort in a progressive ratio test. Previous work showing effects of hippocampal neuroplasticity in tests with less preferred, but not highly preferred, rewards suggests that future investigations of motivation may benefit from testing both weakly and highly preferred rewards. Reward flavorings, which generally had little effect on preference, could be useful in tests requiring distinct but equally preferred rewards.

## Supporting information

S1 File Complete dataset in Excel spreadsheet. (XLSX)

## References

[1] Karlsson R-M, Wang AS, Sonti AN, Cameron HA. Adult neurogenesis affects motivation to obtain weak, but not strong, reward in operant tasks. Hippocampus. 2018;28: 512–522. doi: 10.1002/hipo.22950 29663595 PMC6021202

[2] Kim EW, Phillips BU, Heath CJ, Cho SY, Kim H, Sreedharan J, et al. Optimizing reproducibility of operant testing through reinforcer standardization: identification of key nutritional constituents determining reward strength in touchscreens. Mol Brain. 2017;10: 31. doi: 10.1186/s13041-017-0312-0 28716096 PMC5512767

[3] Phillips BU, Heath CJ, Ossowska Z, Bussey TJ, Saksida LM. Optimisation of cognitive performance in rodent operant (touchscreen) testing: Evaluation and effects of reinforcer strength. Learn Behav. 2017;45: 252–262. doi: 10.3758/s13420-017-0260-7 28205186 PMC5565648

[4] Reilly S. Reinforcement Value of Gustatory Stimuli Determined by Progressive Ratio Performance. Pharmacol Biochem Be. 1999;63: 301–311. doi: 10.1016/s0091-3057(99)00009-x 10371660

[5] Hutsell BA, Newland MC. A quantitative analysis of the effects of qualitatively different reinforcers on fixed ratio responding in inbred strains of mice. Neurobiol Learn Mem. 2013;101: 85–93. doi: 10.1016/j.nlm.2013.01.005 23357283 PMC3649567

[6] Biedermann T, Garlick D, Blaisdell AP. Food choice in the laboratory pigeon. Behav Process. 2012;91: 129–132. doi: 10.1016/j.beproc.2012.06.005 22750307 PMC3404163

[7] Richardson NR, Roberts DC. Progressive ratio schedules in drug self-administration studies in rats: a method to evaluate reinforcing efficacy. Journal of neuroscience methods. 1996;66: 1–11. doi: 10.1016/0165-0270(95)00153-0 8794935

[8] Noonan MA, Bulin SE, Fuller DC, Eisch AJ. Reduction of adult hippocampal neurogenesis confers vulnerability in an animal model of cocaine addiction. The Journal of neuroscience: the official journal of the Society for Neuroscience. 2010;30: 304–315. doi: 10.1523/JNEUROSCI.4256-09.2010 20053911 PMC2844797

[9] Li X, Li W, Wang H, Bayley DL, Cao J, Reed DR, et al. Cats Lack a Sweet Taste Receptor. J Nutrition. 2006;136: 1932S–1934S. doi: 10.1093/jn/136.7.1932s 16772462 PMC2063449

[10] Pinhas A, Aviel M, Koen M, Gurgov S, Acosta V, Israel M, et al. Strain differences in sucrose- and fructose-conditioned flavor preferences in mice. Physiol Behav. 2012;105: 451–459. doi: 10.1016/j.physbeh.2011.09.010 21945373 PMC3225606

[11] Sclafani A. Sucrose motivation in sweet “sensitive” (C57BL/6J) and “subsensitive” (129P3/J) mice measured by progressive ratio licking. Physiol Behav. 2006;87: 734–744. doi: 10.1016/j.physbeh.2006.01.017 16530236

[12] Tenk CM, Felfeli T. Sucrose and fat content significantly affects palatable food consumption in adolescent male and female rats. Appetite. 2017;118: 49–59. doi: 10.1016/j.appet.2017.07.016 28720377

[13] Skjoldager P, Pierre PJ, Mittleman G. Reinforcer Magnitude and Progressive Ratio Responding in the Rat: Effects of Increased Effort, Prefeeding, and Extinction. Learn Motiv. 1993;24: 303–343. doi: 10.1006/lmot.1993.1019

[14] Johnson AW, Gallagher M. Greater effort boosts the affective taste properties of food. Proc Royal Soc B Biological Sci. 2011;278: 1450–1456. doi: 10.1098/rspb.2010.1581 21047860 PMC3081738

[15] Inzlicht M, Shenhav A, Olivola CY. The Effort Paradox: Effort Is Both Costly and Valued. Trends Cogn Sci. 2018;22: 337–349. doi: 10.1016/j.tics.2018.01.007 29477776 PMC6172040

[16] Anselme P. Effort-motivated behavior resolves paradoxes in appetitive conditioning. Behav Process. 2021;193: 104525. doi: 10.1016/j.beproc.2021.104525 34601051

[17] Dayan P. “Liking” as an early and editable draft of long-run affective value. Plos Biol. 2022;20: e3001476. doi: 10.1371/journal.pbio.3001476 34986138 PMC8730425

